# Interspecific variation in the relationship between clutch size, laying date and intensity of urbanization in four species of hole‐nesting birds

**DOI:** 10.1002/ece3.2335

**Published:** 2016-07-25

**Authors:** Marie Vaugoyeau, Frank Adriaensen, Alexandr Artemyev, Jerzy Bańbura, Emilio Barba, Clotilde Biard, Jacques Blondel, Zihad Bouslama, Jean‐Charles Bouvier, Jordi Camprodon, Francesco Cecere, Anne Charmantier, Motti Charter, Mariusz Cichoń, Camillo Cusimano, Dorota Czeszczewik, Virginie Demeyrier, Blandine Doligez, Claire Doutrelant, Anna Dubiec, Marcel Eens, Tapio Eeva, Bruno Faivre, Peter N. Ferns, Jukka T. Forsman, Eduardo García‐del‐Rey, Aya Goldshtein, Anne E. Goodenough, Andrew G. Gosler, Arnaud Grégoire, Lars Gustafsson, Iga Harnist, Ian R. Hartley, Philipp Heeb, Shelley A. Hinsley, Paul Isenmann, Staffan Jacob, Rimvydas Juškaitis, Erkki Korpimäki, Indrikis Krams, Toni Laaksonen, Marcel M. Lambrechts, Bernard Leclercq, Esa Lehikoinen, Olli Loukola, Arne Lundberg, Mark C. Mainwaring, Raivo Mänd, Bruno Massa, Tomasz D. Mazgajski, Santiago Merino, Cezary Mitrus, Mikko Mönkkönen, Xavier Morin, Ruedi G. Nager, Jan‐Åke Nilsson, Sven G. Nilsson, Ana C. Norte, Markku Orell, Philippe Perret, Christopher M. Perrins, Carla S. Pimentel, Rianne Pinxten, Heinz Richner, Hugo Robles, Seppo Rytkönen, Juan Carlos Senar, Janne T. Seppänen, Luis Pascoal da Silva, Tore Slagsvold, Tapio Solonen, Alberto Sorace, Martyn J. Stenning, Piotr Tryjanowski, Mikael von Numers, Wieslaw Walankiewicz, Anders Pape Møller

**Affiliations:** ^1^ Ecologie Systématique EvolutionUniversité Paris‐Sud, CNRS, Agro Paris Tech, Université Paris‐SaclayOrsayFrance; ^2^ Department of BiologyEvolutionary Ecology GroupUniversity of AntwerpAntwerpBelgium; ^3^ Institute of BiologyKarelian Research CentreRussian Academy of SciencesPetrozavodskRussia; ^4^ Department of Experimental Zoology & Evolutionary BiologyUniversity of LodźLodźPoland; ^5^ Terrestrial Vertebrates Research Unit “Cavanilles”Institute of Biodiversity and Evolutionary BiologyUniversity of ValenciaPaternaSpain; ^6^ Université Pierre et Marie CurieSorbonne universitésUPMC Univ Paris 06, UPEC, Paris 7CNRS, INRA, IRD, Institut d’Écologie et des Sciences de l'Environnement de ParisParisFrance; ^7^ Centre d'Ecologie Fonctionnelle et Evolutive, Campus CNRSMontpellierFrance; ^8^ Research Laboratory “Ecology of Terrestrial and Aquatic Systems”University Badji MokhtarAnnabaAlgeria; ^9^ INRAPlantes et Systèmes de culture HorticolesAvignonFrance; ^10^ Àrea de BiodiversitatGrup de Biologia de la ConservacióCentre Tecnològic Forestal de CatalunyaSolsonaSpain; ^11^ Strada BineAcquanegra sul Chiese (MM)Italy; ^12^ University of HaifaHaifaIsrael; ^13^ Society for the Protection of NatureUniversity of LausanneLausanneSwitzerland; ^14^ Institute of Environmental ScienceJagiellonian UniversityKrakowPoland; ^15^ Department of Agriculture and Forest SciencesUniversità di PalermoPalermoItaly; ^16^ Department of ZoologyFaculty of Natural ScienceSiedlce University of Natural Sciences and HumanitiesSiedlcePoland; ^17^ Department of Biometry & Evolutionary BiologyUniversity of Lyon 1VilleurbanneFrance; ^18^ Museum and Institute of ZoologyPolish Academy of SciencesWarsawPoland; ^19^ Department of BiologyBehavioural Ecology and Ecophysiology GroupUniversity of AntwerpAntwerpBelgium; ^20^ Section of EcologyDepartment of BiologyUniversity of TurkuTurkuFinland; ^21^ BioGéoSciencesUniversité de BourgogneDijonFrance; ^22^ School of BioscienceCardiff UniversityCardiffUK; ^23^ Department of EcologyUniversity of OuluOuluFinland; ^24^ Departamento de EcologíaFacultad de BiologíaUniversidad de La Laguna, San Cristóbal de La LagunaTenerife Canary IslandsSpain; ^25^ Tel‐Aviv UniversityTel‐AvivIsrael; ^26^ Department of Natural and Social SciencesUniversity of GloucestershireGloucestershireUK; ^27^ Department of ZoologyEdward Grey Institute of Field Ornithology & Institute of Human SciencesOxfordUK; ^28^ Department of Animal EcologyEvolutionary Biology CentreUppsala UniversityUppsalaSweden; ^29^ Lancaster Environment CentreLancaster UniversityLancasterUK; ^30^ Laboratoire Évolution & Diversité BiologiqueUPS Toulouse IIIToulouseFrance; ^31^ CEH WallingfordMaclean BuildingWallingfordOxfordshireUK; ^32^ Institute of Ecology of Nature Research CentreAkademijos 2VilniusLithuania; ^33^ Institute of Ecology & Earth SciencesUniversity of TartuTartuEstonia; ^34^ Crx. St. PierreFleurey Sur OucheFrance; ^35^ Departamento de Ecología Evolutiva Museo Nacional de Ciencias NaturalesAgencia Estatal Consejo Superior de Investigaciones CientíficasMadridSpain; ^36^ Department of ZoologyRzeszów UniversityRzeszówPoland; ^37^ Department of Biological and Environmental SciencesUniversity of JyväskyläJyväskyläFinland; ^38^ Institute of Biodiversity, Animal Health & Comparative MedicineUniversity of GlasgowGlasgowUK; ^39^ Animal EcologyLund UniversityLundSweden; ^40^ Department of Life SciencesInstitute of Marine ResearchUniversity of CoimbraCoimbraPortugal; ^41^ Department of Life SciencesMARE ‐ Marine and Environmental Sciences CentreUniversity of CoimbraCoimbraPortugal; ^42^ Centro de Estudos FlorestaisInstituto Superior de AgronomiaUniversity of LisbonLisbonPortugal; ^43^ Didactica Research UnitFaculty of Social SciencesUniversity of AntwerpAntwerpBelgium; ^44^ Institute of Ecology & Evolution (IEE)University of BernBernSwitzerland; ^45^ Evolutionary Biology Group (GIBE)Falculty of SciencesUniversity of A CoruñaA CoruñaSpain; ^46^ Unidad Asociada CSIC de Ecología Evolutiva y de la ConductaNat‐Museu de Ciències Naturals de BarcelonaBarcelonaSpain; ^47^ Department of BiosciencesUniversity of OsloOsloNorway; ^48^ Luontotutkimus Solonen OyHelsinkiFinland; ^49^ SROPURomeItaly; ^50^ School of Life SciencesUniversity of SussexEast SussexUK; ^51^ Institute of ZoologyPoznan University of Life SciencesPoznańPoland; ^52^ Environmental and Marine BiologyÅbo Akademi UniversityÅboFinland

**Keywords:** Breeding phenology, orthophotograph, passerine birds, population dynamics, urban heat island effect

## Abstract

The increase in size of human populations in urban and agricultural areas has resulted in considerable habitat conversion globally. Such anthropogenic areas have specific environmental characteristics, which influence the physiology, life history, and population dynamics of plants and animals. For example, the date of bud burst is advanced in urban compared to nearby natural areas. In some birds, breeding success is determined by synchrony between timing of breeding and peak food abundance. Pertinently, caterpillars are an important food source for the nestlings of many bird species, and their abundance is influenced by environmental factors such as temperature and date of bud burst. Higher temperatures and advanced date of bud burst in urban areas could advance peak caterpillar abundance and thus affect breeding phenology of birds. In order to test whether laying date advance and clutch sizes decrease with the intensity of urbanization, we analyzed the timing of breeding and clutch size in relation to intensity of urbanization as a measure of human impact in 199 nest box plots across Europe, North Africa, and the Middle East (i.e., the Western Palearctic) for four species of hole‐nesters: blue tits (*Cyanistes caeruleus*), great tits (*Parus major*), collared flycatchers (*Ficedula albicollis*), and pied flycatchers (*Ficedula hypoleuca*). Meanwhile, we estimated the intensity of urbanization as the density of buildings surrounding study plots measured on orthophotographs. For the four study species, the intensity of urbanization was not correlated with laying date. Clutch size in blue and great tits does not seem affected by the intensity of urbanization, while in collared and pied flycatchers it decreased with increasing intensity of urbanization. This is the first large‐scale study showing a species‐specific major correlation between intensity of urbanization and the ecology of breeding. The underlying mechanisms for the relationships between life history and urbanization remain to be determined. We propose that effects of food abundance or quality, temperature, noise, pollution, or disturbance by humans may on their own or in combination affect laying date and/or clutch size.

## Introduction

The apparent dichotomy between urban and rural areas is usually used to analyze the impact of urban habitats on populations, although this definition does not consider that rural areas may also be urbanized when compared to truly natural habitats. Indeed, Pickett et al. (2011) defined urbanized areas as those where people live in high densities and also where infrastructures such as roads or bridges as well as buildings cover most of the surface. Urbanized areas influence climate and soil characteristics with impacts on ecosystems (Pickett et al. 2011). Temperatures are generally higher in cities than in neighboring rural or natural areas, phenomenon known as “heat island effect” (Escourrou 1990; Pachauri and Reisinger 2008; Stocker et al. 2013). These temperature increases are influenced by urban human population density (Gaston 2010; Pickett et al. 2011; Susca et al. 2011). High human population density also causes socio‐politico‐economic pressures on ecosystems that provide services such as food, raw materials, recreational values and decontaminated water and atmosphere for human populations (Grimm et al. 2008; Gaston 2010; Pickett et al. 2011), although urban areas also support animal and plant species (Aronson et al. 2014).

Urbanization characteristics influence ecosystems at all levels, from individuals to communities, depending on the systematic group considered. For example, humans greatly modify plant communities in parks and gardens across cities and often urban communities are dominated by non‐native plants that have lower insect populations (Pickett et al. 2011). A lower diversity of insects, amphibians, and reptiles occurs in urban compared to rural areas, and the abundance of domestic animals such as cats and dogs increases with human density (Bol'shakov et al. 2001; Gil and Brumm 2013; Johnson et al. 2013; Vittoz et al. 2013). In birds, generalists are more predominant than specialists in urban areas (Blair 1996; Devictor et al. 2008; Shwartz et al. 2008; Sorace and Gustin 2009; Huste and Boulinier 2011). Moreover, the behavior of animals, but especially also of birds, is influenced by environmental urban characteristics, for example, noisy backgrounds and/or buildings influence intra‐ and interspecific communication such as acoustic detection of predators and conspecifics (Brumm 2004; Barber et al. 2010; Snell‐Rood 2012; Slabbekoorn 2013), and artificial light during night perturbs circadian and annual rhythms affecting sleep and timing of breeding (Small and Elvidge 2011; Dominoni et al. 2014; Fonken and Nelson 2014; Raap et al. 2015). Moreover, a previous quantitative review demonstrated a significant advance in laying dates in urban areas for five bird species (including great tits) and a delay for one of a total of ten avian species considered (Chamberlain et al. 2009).

A mismatch between phenology and suitable timing of migration or breeding may reduce individual fitness and affect population dynamics in birds (Visser et al. 2004, 2012). Hatching date is constrained by laying date, clutch size and incubation date (Godfray et al. 1991; Visser et al. 2004). Synchrony between individual behavior and suitable timing of breeding is determined by environmental clues (Parmesan 2006; Visser et al. 2006; Sih et al. 2011) such as temperature (Both et al. 2004; Charmantier et al. 2008; Naef‐Daenzer et al. 2012), light (Dominoni et al. 2013), and date of bud burst (Visser et al. 2012). Moreover, global temperature increases in early spring have advanced the phenology of birds over the last few decades (Both and Visser 2001; Both et al. 2004; Visser et al. 2006; Both and te Marvelde 2007; Møller et al. 2010; Porlier et al. 2012; Charmantier and Gienapp 2014; Dunn and Møller 2014). As urban areas are usually warmer than the surrounding rural areas, breeding in urban areas could be advanced by higher ambient temperatures (Escourrou 1990; Pachauri and Reisinger 2008; Stocker et al. 2013) and/or by artificial night light (Small and Elvidge 2011; Dominoni et al. 2014; Fonken and Nelson 2014), but also by more intensive feeding of birds (Stenning 1995; Robb et al. 2008a,b). These modifications may make urban areas become habitable to migrant birds arriving at their breeding grounds slightly earlier than nearby rural areas, which may be earlier than more natural areas (Tryjanowski et al. 2013; Dunn and Møller 2014).

In rural and natural habitats, egg laying of birds is delayed by cold prelaying temperatures (Charmantier et al. 2008; Visser et al. 2009; Naef‐Daenzer et al. 2012; Schaper et al. 2012; Chmielewski et al. 2013; Vatka et al. 2014), at high latitudes (Mainwaring et al. 2012; Ruffino et al. 2014), or when bud burst date is delayed (Naef‐Daenzer et al. 2012). Moreover, the both effects of latitude (Mainwaring et al. 2012) and bud burst (Schaper et al. 2011; Visser et al. 2012) seem to be related to temperature effects. In migratory species, laying date is mainly determined by arrival date, which in turn is advanced by global temperature increases especially in northerly populations (Walther et al. 2002; Both and te Marvelde 2007; Pulido 2007) even if laying date and arrival date of some migratory species are more poorly correlated than in others (Laaksonen et al. 2006) or in southern populations (Goodenough et al. 2011). Thus, variation in temperature and environmental conditions more broadly are the determinants of breeding phenology.

Previous studies of the effects of urbanization on avian life history variables have often relied on a single or a couple of populations (Hõrak et al. 2002; Isaksson and Andersson 2007; Chamberlain et al. 2009; Brahmia et al. 2013), which does not allow for generalizations or inferences regarding spatial heterogeneity. The aim of this study was to relate breeding ecology to the intensity of local urbanization, a proxy of density and influence of humans on ecosystem, by analyzing laying dates and clutch sizes in four species of hole‐nesting passerine birds, in relation to the degree of urbanization across Europe, North Africa, and the Middle East. We used hole‐nesting birds as a model system because the breeding phenology is easier to follow than in open‐cup nesters, and, therefore, they are routinely studied by scientists and amateurs across the Western Palearctic. This study was based on almost 200 study plots with a total of almost 80,000 reproductive events. Such extensive data are unavailable for other species of birds, but also for other organisms. These extensive data facilitated the current study. A decrease in clutch size is one option to advance hatching date (Visser et al. 2004), but could also be a response to environmental conditions in urban area as food quality, human disturbance or cat predation (Gil and Brumm 2013) or population density (Krebs 1970; Stenning et al. 1988). If intensity of urbanization did not influence laying date, we analyzed the relationship between clutch size and urban intensity with laying date as fixed factor. As ambient temperatures are higher at lower latitudes in Europe (Schönwiese and Rapp 2013), and temperature seems to be one of the main determinants of avian breeding phenology, we analyzed the interaction between latitude and intensity of urbanization on laying date. We expected a stronger impact of urbanization on tits compared to flycatchers. Indeed, flycatchers are sub‐Saharan migrants that spend less time in urban areas and a laying date that is influenced by arrival date in northern populations, while laying date of tits is mainly determined by local conditions at the breeding sites (Pearson and Lack 1992; Both et al. 2006). Finally, laying date may also vary with habitat structure (Van Balen 1973; Mänd et al. 2005; Arriero et al. 2006; Mizuta 2006) or the presence of predators (Lank and Ydenberg 2003; Sergio et al. 2007). Larger and deeper nest boxes (Mertens 1977; Van Balen 1984; Summers and Taylor 1996) and concrete boxes (O'Connor 1978) offer better thermal isolation and better protection from predation. Thus, the influence of dominant habitat, nest floor surface, and nest box material on laying date and clutch size were also analyzed.

## Material and Methods

### Study species

Blue tits (*Cyanistes caeruleus*), great tits (*Parus major*), pied flycatchers (*Ficedula hypoleuca*), and collared flycatchers (*Ficedula albicollis*) are all small insectivorous passerine birds that breed commonly in nest boxes in large parts of Europe. The two species of tits are residents or partial short‐distance migrants depending on their population (Nowakowski and Vähätalo 2003), while the two flycatchers species are both migratory and spend the winter months in sub‐Saharan West Africa.

### Data

Annual mean breeding dates, clutch sizes, and sample sizes of first clutches derive from an exhaustive attempt to obtain information from populations across Europe, North Africa, and the Middle East (Fig. S1; Møller et al. 2014). We used mean laying date per population and year of (1) 101 study populations of blue tits, with a total of 1127 study years for laying date, and 1124 study years for clutch size; (2) 138 study populations of great tits, 1439 study years for laying date, and 1436 for clutch size; (3) 66 study populations of collared flycatchers, with a total of 592 study years for both parameters; and (4) 23 populations of pied flycatchers, with a total of 259 study years. Information on latitude, longitude, altitude, mean study year, species, dominant breeding habitat (coniferous, deciduous, evergreen, or mixed forest), nest box floor area, and nest box material (wood or concrete) for all study plots were provided by scientists or reported in a previous publication (Møller et al. 2014). Borders and numbers of nest boxes depended on study plot as determined by researchers who monitored the populations.

The increase in the density of buildings is known to correlate with the increase in the total number of individual birds (Hedblom and Söderström 2010), advanced laying date (Shustack and Rodewald 2010), and increased breeding success (Ryder et al. 2010; Hedblom and Söderström 2012). All study plots were classified by each researcher who followed a given population as either rural or urban without single criterion (see Supporting information). This dichotomy is commonly used, but inadequate when quantifying human influence because rural areas include agricultural, and natural habitats and urban areas include parks and gardens with mature trees. Therefore, we recorded an estimate of the density of buildings using information from ArcGIS Earthstar Geographics for each of the study plots (*N* = 199). An index of the “Intensity of urbanization” was obtained by dividing the number of buildings by the area of the study site (see next paragraph) followed by log‐transformation of density of buildings adding a constant of one to avoid values of zero and to normalize the data. We counted the number of roofs of each building as the number of roofs with one color and one direction, while L‐shaped buildings were counted as two roofs. We used the density of buildings and not the cover by roads or buildings because these descriptive variables are strongly positively correlated (Shustack and Rodewald 2010). The percentage of built‐up area within 1‐km circles was strongly positively correlated with the number of roofs (*F*_1,32_ = 81.24, *P *<* *0.01), and previous studies have shown that it is the density of buildings that is correlated with population density (Brumm 2004; Barber et al. 2010; Snell‐Rood 2012; Slabbekoorn 2013). Study plot coordinates were in the form 12.12345°N, 12.12345°E for 160 study plots and in the from 12.12°N, 12.12°E for 39 study plots where the scientist was inaccessible either due to retirement or death, and it was thus impossible to provide more precise coordinates (see Table S1). Analyses were repeated without these imprecise coordinates for study plots, but we found qualitatively similar results.

To estimate the effect of measurement scale, we analyzed the intensity of urbanization in subsamples of 34 of 199 study plots (all study plots classified as urban [*N* = 14] and 20 randomly selected from 185 plots classified as rural plots by scientists [see Table S1]) within a radius of 200, 500, and 1000 m from the centre of each study plot, by visually counting the number of buildings on digital orthophotographs (Shustack and Rodewald 2010). The density of buildings was measured at the scale that allowed identification of different roofs, depending on the orthophotographs available. We used a radius of 200 and 500 m, respectively, because they were similar to the size of most study plots and 1000 m to validate the method at the level of study plots. Use of a buffer circular area around study plots provided conservative estimates of intensity of urbanization among study plots. However, the intensity of urbanization at the three distances was highly repeatable (200–500 m, *F*_33,34_ = 8.20, *P *<* *0.01, intraclass correlation coefficient = 0.78 (Lessells and Boag 1987) and 200–1000 m, *F*_33,34_ = 3.30, *P *<* *0.01, intraclass correlation coefficient = 0.54). Therefore, we only used an estimate of the intensity of urbanization (log[number of building/area of study + 1]) for a radius of 200 m in the subsequent analyses; that is, an intensity of urbanization was recorded in each of the study plots in 2015 (*N *=* *199). Our indicator of intensity of urbanization was on average 59 buildings/km^2^ (SE = 4, range 0–1305 buildings/km², *N* = 199). We found a strong positive relationship between the binomial score of urbanization provided by scientists and intensity of urbanization near the nest box plots for all study plots (Student *t*‐test: *t*_df_ = −6.26_14.19_, *P *<* *0.0001, Mean ± SE log‐transformed index = 0.47 ± 0.06 and 2.15 ± 0.26 for rural (*N* = 185) and urban (*N* = 14) areas, respectively, see Fig. S1A). Moreover, intensity of urbanization was negatively related to CORINE land cover code (Kendall rank order test: *τ* = −0.46, *t*_df_ = −7.11_189_, *P *<* *0.0001, *N* = 191, see Fig. S1B). CORINE land cover code assessed the land cover in classes (agricultural areas, artificial surfaces or forests areas), with values decreasing with degree of anthropogenization of areas. Theses codes were available for most areas of Europe, but not North Africa and Middle East. Intensity of urbanization in agricultural areas was intermediate between that in urban and natural sites.

For some species, latitude is correlated with laying date (Mainwaring et al. 2012; Ruffino et al. 2014), and to take this into account, geographic coordinates of study plots were used in the models. Latitude and longitude of the study plots were positively correlated (Pearson *r *=* *0.48, *t*_197_ = 7.64, *P *<* *0.01, Fig. S2), although not causing problems of collinearity (based on correlograms; Dormann et al. 2007). The interaction between longitude and latitude and quadratic terms for latitude and longitude were entered in models to account for nonlinear relationships and spatial autocorrelation (Legendre 1993). There was no autocorrelation in model residuals (Moran test; Dormann et al. 2007).

Collared flycatchers only used wooden nest boxes, while the two species of flycatchers were absent from evergreen habitats.

### Statistical analyses

All statistical analyses were performed in R v. 3.2.0 (R Core Team 2015). Explanatory variables were correlated, but coefficients were small (see Table S2). We used linear mixed models and backward elimination of factors using Akaike's information criterion (AIC) to select the best predictive model to explain variation in laying date (package nlme, function lme, method REML and package car, function Anova, type III). The initial model included the three‐way interaction (latitude × species × intensity of urbanization), two‐way interactions (latitude × species, species × intensity of urbanization, latitude × intensity of urbanization and latitude × longitude), study plot as random factor and intensity of urbanization, log‐transformed altitude, latitude, latitude squared, longitude, longitude squared, nest floor surface, material of nest box and dominant habitat as fixed factors. As the three‐way interaction was significant (see Table S3), models were subsequently developed and performed for each of the four species separately.

In the second part, for species for which the interaction between latitude and intensity of urbanization and the main effect of intensity of urbanization were not significant, the initial model to explain variation in clutch size included two‐way interactions (latitude  × intensity of urbanization and latitude × longitude), study plot as random factor and intensity of urbanization, laying date, log‐transformed altitude, latitude, latitude squared, longitude, longitude squared, nest floor surface, material of nest box, and dominant habitat as fixed factors. Altitude was log‐transformed to avoid residuals of models that deviated from normal distributions. As discussed in detail above, we used linear mixed models and backward elimination of factors using AIC (package nlme, function lme, method REML and package car, function Anova, type III).

Several nests were followed in each plot and each year (blue tits: from 1 to 154 nests, mean ± SE = 17 ± 22 nests per year and per plot/great tits: from 1 to 210 nests, mean ± SE = 20 ± 24 nests per year and per plot/collared flycatchers: from 1 to 159 nests, mean ± SE = 26 ± 19 nests per year and per plot/pied flycatchers: from 1 to 189 nests, mean ± SE = 50 ± 35 nests per year and per plot). The number of nests per year and per study plot was used for weighting each data point, thereby assuring that each observation contributed to the models relative to the level of sampling (Draper and Smith 1998; Kutner et al. 2004). Likelihood ratio tests (LRT) were manually calculated for the random effect of study plot. No residuals of final models deviated from normal distributions.

## Results

### Laying date

**Figure 1:**
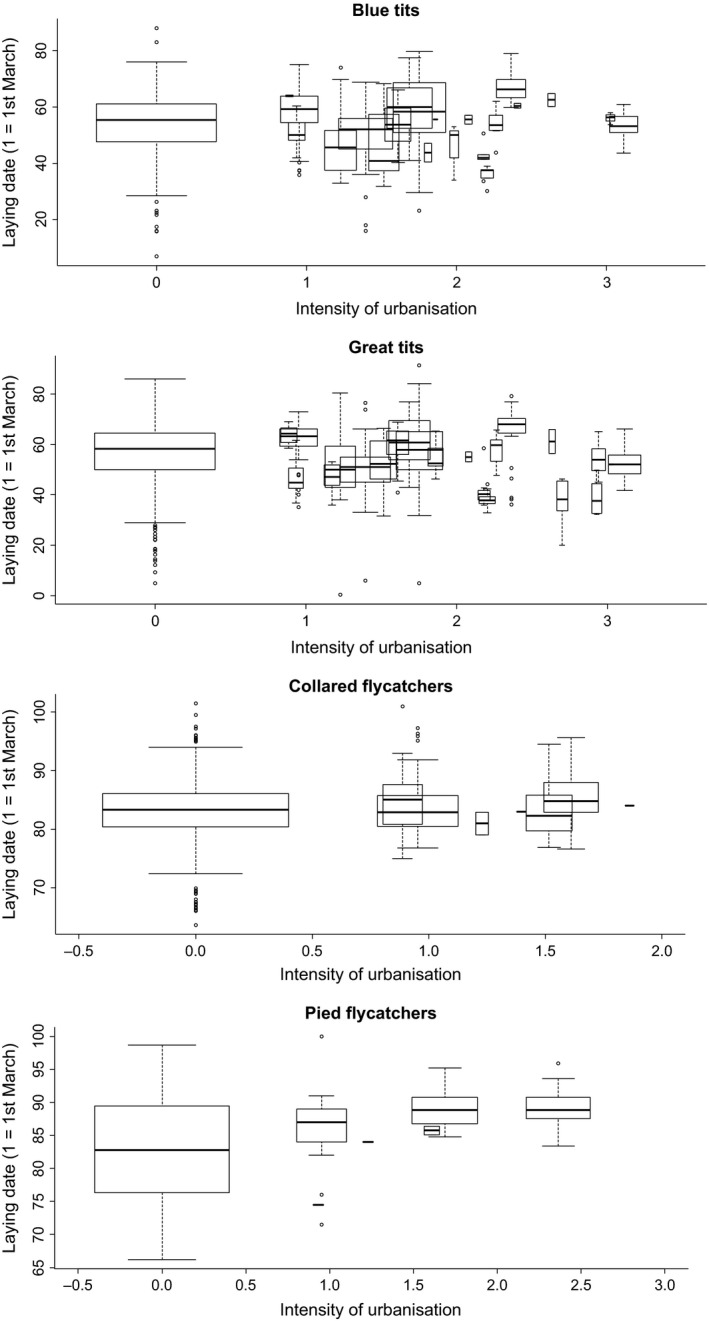
Box plots of laying date in relation to intensity of urbanization in four passerine bird species in Europe, North Africa, and the Middle East. Box plots show medians, quartiles, 5‐ and 95‐percentiles, and extreme values. Width of box plots reflects sample size (study populations/total number of years: 100/1125, 138/1439, 66/592, and 23/259 for blue tit, great tit, collared flycatcher, and pied flycatcher, respectively). Intensity of urbanization was estimated as the local density of buildings per km² and log‐transformed.

Box plots of laying dates for the four species across a gradient of “intensity of urbanization” are shown in Figure 1. For all four species, the interaction between latitude and intensity of urbanization, the main effect of intensity of urbanization, and altitude were not significant, while laying date advanced significantly over years (Table 1).

**Table 1:** Results of linear models investigating variation in laying date as a function of two‐way interactions (latitude × intensity of urbanization and latitude × longitude), study plot (random factor), intensity of urbanization, altitude (log‐transformed), latitude, latitude squared, longitude and longitude squared, year, nest floor area, nest box material, and dominant habitat (fixed factors)

Species	Blue tit			Great tit			Collared flycatcher			Pied flycatcher		
Study populations	101			138			66			23		
Total number of study years	1127			1439			592			259		
Final (initial) model AIC	7837.31 (*7840.73*)			10,513.13 (*10,526.64*)			3875.78 (*3879.74*)			1764.44 (*1795.32*)		
	*F*_df_	*P*	Estimate ± SE	*F*_df_	*P*	Estimate ± SE	*F*_df_	*P*	Estimate ± SE	*F*_df_	*P*	Estimate ± SE
Intensity of urbanization	0.78_1,98_	0.39	−1.16 ± 1.32	*0.21*_*1,132*_	*0.65*	*3.01 ± 6.61*	0.15_1,60_	0.70	269.66 ± 690.06	*0.27*_*1,18*_	*0.60*	*14.86 ± 28.47*
Latitude	9.87_1,1014_	**<0.01**	−9.64 ± 3.07	2.12_1,134_	0.15	−3.93 ± 2.70	0.96_1,60_	0.33	8.27 ± 8.44	192.14_1,234_	**<0.01**	1.74 ± 0.13
Latitude^2^	11.40_1,1014_	**<0.01**	0.11 ± 0.03	2.42_1,134_	0.12	0.04 ± 0.03	*0.21*_*1,56*_	*0.65*	−*0.39 *±* 0.84*	*0.04*_*1,227*_	*0.84*	−*0.02 ± 0.10*
Longitude	6.94_1,1014_	**<0.01**	3.61 ± 1.37	2.53_1,134_	0.11	−0.89 ± 0.56	0.70_1,60_	0.40	18.71 ± 22.35	*0.01*_*1,227*_	*0.92*	*0.21 ± 3.46*
Longitude^2^	5.70_1,1014_	**0.02**	0.06 ± 0.03	23.50_1,134_	<**0.01**	−0.05 ± 0.01	*0.10*_*1,56*_	*0.75*	−*0.49 *±* 1.52*	*0.01*_*1,227*_	*0.92*	−*0.01 ± 0.02*
Year	161.99_1,14014_	**<0.01**	−0.19 ± 0.02	36.00_1,1292_	**<0.01**	−0.09 ± 0.01	34.83_1,524_	**<0.01**	−0.15 ± 0.03	24.51_1,234_	**<0.01**	−0.15 ± 0.03
Altitude (log)	0.11_1,1014_	0.74	0.66 ± 1.95	1.48_1,1292_	0.29	1.47 ± 1.40	0.74_1,524_	0.39	−3.24 ± 3.78	*1.55*_*1,18*_	*0.21*	−*2.05 ± 1.65*
Nest floor area	6.37_1,1014_	**0.01**	−0.05 ± 0.02	*0.30*_*1,1291*_	*0.59*	*0.01 *±* 0.01*	*0.31*_*1,523*_	*0.58*	−*0.04 *±* 0.07*	*1.54*_*1,227*_	*0.22*	−*0.02 ± 0.01*
Nest box material	1.25_1,1014_	0.26		1.16_1,1292_	0.28		–	–		4.72_1,21_	**0.03**	
Wood			−3.59 ± 3.21			2.48 ± 2.31			–			4.45 ± 2.05
Dominant habitat	60.56_3,1014_	**<0.01**		12.65_1,1292_	**<0.01**		*0.04*_*2,56*_	*0.98*		*0.06*_*2,227*_	*0.97*	
Deciduous			−13.75 ± 2.00			−1.44 ± 1.98			−*0.33 ± 1.60*			−*0.31 ± 2.21*
Evergreen			−10.26 ± 2.08			5.61 ± 2.75			*–*			*–*
Mixed			−4.36 ± 1.01			−2.56 ± 1.26			−*0.41 ± 3.50*			−*0.33 ± 1.35*
*Intensity of urbanization × latitude*	*0.10*_*1,1013*_	*0.76*	*0.06 ± 0.19*	*0.25*_*1,1291*_	*0.62*	−*0.07 *±* 0.13*	0.15_1,60_	0.70	−4.73 ± 12.10	*0.25*_*1,18*_	*0.62*	−*0.23 *±* 0.47*
Latitude × longitude	7.40_1,1014_	**<0.01**	−0.09 ± 0.03	13.22_1,134_	**<0.01**	0.04 ± 0.01	0.74_1,60_	0.39	−0.38 ± 0.44	*0.01*_*1,227*_	*0.97*	−*0.01 *±* 0.07*
Study plot	LRT: −28.41	0.50		LRT: 404.70	**<0.01**		LRT: 523.46	**<0.01**		LRT: 351.304	**<0.01**	

Initial values of variables that were not retained in the final models are presented in italics, and significant *P*‐values in the final models are shown in bold on a gray background.

In blue tits, laying date varied with nest box floor area and study plot coordinates, and differed significantly among habitats (Table 1). In great tits, laying date varied with study plot coordinates, and it differed among habitats (Table 1). In collared flycatchers, laying date was correlated with study plot coordinates, nest box floor area, and habitat (Table 1). In pied flycatchers, laying date varied with study plot coordinates and was earlier in wooden nest boxes compared to concrete boxes (Table 1).

### Clutch size

**Figure 2:**
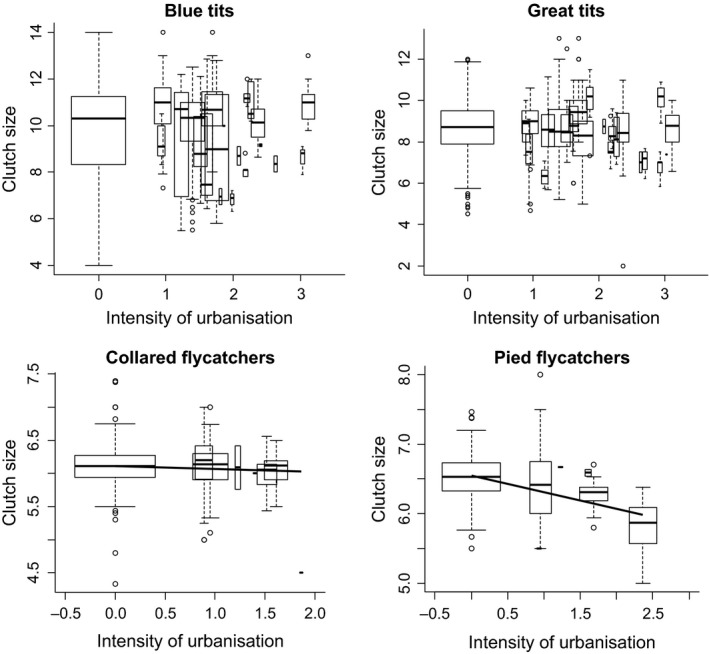
Box plots of clutch size in relation to intensity of urbanization in four passerine bird species in Europe, North Africa, and the Middle East. Box plots show medians, quartiles, 5‐ and 95‐percentiles, and extreme values. Width of box plot reflects sample size (study populations/total number of years: 100/1122, 138/1436, 66/592, and 23/259 for blue tit, great tit, collared flycatcher, and pied flycatcher, respectively). Lines are the linear regression. Intensity of urbanization was estimated as the local density of buildings per km² and log‐transformed.

Box plots of clutch size for the four species across a gradient of “intensity of urbanization” are shown in Figure 2. For all four species, the interaction between latitude and intensity of urbanization and altitude were not significant, and clutch sizes did not differ between habitats but decreased with laying date (Table 2).

**Table 2:** Results of linear models investigating variation in clutch size as a function of two‐way interactions (latitude × intensity of urbanization and latitude × longitude), study plot (random factor), intensity of urbanization, laying date, altitude (log‐transformed), latitude, latitude squared, longitude and longitude squared, year, nest floor area, nest box material, and dominant habitat (fixed factors)

Species	Blue tit			Great tit			Collared flycatcher			Pied flycatcher		
Study populations	101			138			66			23		
Total number of study years	1124			1436			592			259		
Final (initial) model AIC	3911.17 (*3958.37*)			5611.25 (*5645.29*)			956.86 (*993.03*)			640.50 (*712.91*)		
	*F*_df_	*P*	Estimate ± SE	*F*_df_	*P*	Estimate ± SE	*F*_df_	*P*	Estimate ± SE	*F*_df_	*P*	Estimate ± SE
Intensity of urbanization	*0.02*_*1,98*_	*0.88*	−*0.13 ± 0.88*	*0.05*_*1,132*_	*0.82*	*0.17 ± 0.75*	9.66_1,64_	**<0.01**	−0.24 ± 0.08	4.18_1,20_	**0.04**	−0.23 ± 0.11
Laying date	46.15_1,1018_	**<0.01**	−0.033 ± 0.005	69.69_1,1294_	**<0.01**	−0.039 ± 0.005	101.37_1,523_	**<0.01**	−8.65 ± 4.73	97.89_1,234_	**<0.01**	−0.07 ± 0.01
Latitude	8.90_1,1018_	**<0.01**	0.70 ± 0.23	7.46_1,134_	**<0.01**	0.82 ± 0.30	*1.66*_*1,60*_	*0.20*	*9.00 ± 6.99*	10.82_1,234_	**<0.01**	0.09 ± 0.03
Latitude^2^	4.68_1,1018_	**0.03**	−0.05 ± 0.002	6.76_1,134_	**<0.01**	−0.008 ± 0.003	*1.73*_*1,56*_	*0.19*	−*0.13 ± 0.10*	*0.01*_*1,226*_	*0.96*	−*0.01 ± 0.01*
Longitude	*1.37*_*1,1010*_	*0.24*	*0.17 ± 0.15*	1.41_1,134_	0.24	−0.08 ± 0.06	*0.78*_*1,60*_	*0.38*	−*5.10 ± 5.76*	*1.07*_*1,226*_	*0.30*	*0.45 ± 0.43*
Longitude^2^	*2.75*_*1,1010*_	*0.10*	*0.004 ± 0.003*	0.53_1,134_	0.28	−0.01 ± 0.01	*2.11*_*1,56*_	*0.15*	−*0.26 *±* 0.18*	*0.89*_*1,226*_	*0.35*	*0.01 ± 0.01*
Year	29.04_1,14018_	**<0.01**	−0.015 ± 0.003	59.84_1,1294_	**<0.01**	−0.021 ± 0.003	14.72_1,523_	**<0.01**	0.009 ± 0.002	*1.53*_*1,226*_	*0.22*	*0.01 ± 0.01*
Altitude (log)	*1.68*_*1,1010*_	*0.19*	−*0.26 ± 0.20*	*1.49*_*1,1288*_	*0.22*	*0.21 ± 0.18*	3.16_1,523_	0.08	−0.20 ± 0.11	*0.09*_*1,18*_	*0.77*	−*0.08 ± 0.25*
Nest floor area	12.64_1,1018_	**<0.01**	−0.010 ± 0.003	*0.59*_*1,1288*_	*0.44*	*0.01 *±* 0.01*	*0.89*_*1,523*_	*0.35*	*0.01 *±* 0.01*	*2.37*_*1,226*_	*0.12*	−*0.01 ± 0.01*
Nest box material	7.44_1,1018_	**<0.01**		*0.93*_*1,1288*_	*0.34*		–	–		15.49_1,20_	**<0.01**	
Wood			0.83 ± 0.30			*0.28 ± 0.29*		–	–			1.26 ± 0.32
*Dominant habitat*	*4.55*_*3,1010*_	*0.21*		*2.59*_*1,1288*_	*0.46*		*0.01*_*2,56*_	*0.99*		*1.27*_*2,226*_	*0.53*	
*Deciduous*			*0.43 ± 0.25*			*0.16 ± 0.26*			*0.01 ± 0.23*			*0.28 ± 0.27*
*Evergreen*			*0.21 ± 0.30*			−*0.22 ± 0.36*			*–*			*–*
*Mixed*			*0.33 ± 0.18*			*0.22 ± 0.22*			−*0.01 ± 0.42*			*0.15 ± 0.16*
*Intensity of urbanization* × *latitude*	*0.01*_*1,1010*_	*0.96*	−*0.01 ± 0.02*	*0.10*_*1,132*_	*0.76*	−*0.01 *±* 0.01*	*0.01*_*1,56*_	*0.97*	*0.06 ± 1.56*	*0.46*_*1,18*_	*0.50*	−*0.04 *±* 0.06*
Latitude × longitude	*1.83*_*1,1010*_	*0.18*	−*0.005 ± 0.004*	6.92_1,1294_	**<0.01**	0.003 ± 0.001	*1.63*_*1,56*_	*0.20*	*0.30 ± 0.23*	*1.12*_*1,226*_	*0.29*	−*0.01 *±* 0.01*
Study plot	LRT: 790.62	**<0.01**		LRT: 1384.18	**<0.01**		LRT: 754.05	**<0.01**		LRT: 496.76	**<0.01**	

Initial values of variables that were not retained in the final models are presented in italics, and significant *P*‐values in the final models are shown in bold on a gray background.

In blue tits, clutch size did not vary significantly with the intensity of urbanization, but decreased across years, varied with nest box floor area, nest box material and study plot coordinates (Table 2). In great tits, clutch size did not vary significantly with intensity of urbanization, but decreased across years and varied with study plot (Table 2). In collared flycatchers, clutch size decreased with intensity of urbanization and increased across years (Table 2). In pied flycatchers, clutch size decreased with intensity of urbanization, varied with study plot coordinates and was larger in wooden than in concrete nest boxes (Table 2).

## Discussion

We analyzed the breeding ecology of four species of passerine birds in nest boxes in relation to the intensity of urbanization across Europe, North Africa, and the Middle East. Nest box characteristics, habitat, and geographic location were included in the models to account for potentially confounding environmental effects other than that of intensity of urbanization. To our knowledge, this is the first large‐scale study of the relationship between intensity of urbanization and avian breeding ecology. The intensity of urbanization was not correlated with laying date in the four species, while clutch sizes decreased with increasing intensity of urbanization in both collared and pied flycatchers.

In all four species, our large‐scale analysis confirmed correlations between laying date, clutch size, and various environmental factors which have previously been demonstrated in single‐specific studies (Van Balen 1973, 1984; Mertens 1977; O'Connor 1978; Pearson and Lack 1992; Summers and Taylor 1996; Both and Visser 2001; Ahola et al. 2004; Both et al. 2004, 2006; Mänd et al. 2005; Arriero et al. 2006; Mizuta 2006; Both and te Marvelde 2007; Charmantier et al. 2008; Magi et al. 2009; Sisask et al. 2010; Mainwaring et al. 2012; Chmielewski et al. 2013; Charmantier and Gienapp 2014; Møller et al. 2014; Ruffino et al. 2014; Vatka et al. 2014). Thus, we focus the remainder of the discussion on the correlation between intensity of urbanization and breeding ecology.

Urbanization is an ongoing process that has intensified over time and differs among countries. Indeed, temporal changes in urbanization varied between 0.1% per year in the Netherlands measured in 1992 (WRR 1992) to more than 2% per year in France between 2000 and 2010 (Clanché and Rascol 2011). With these differences in rate of urbanization, it was difficult to take temporal change in urbanization into account. In order to verify the validity of our measure of intensity of urbanization among years, we only analyzed data collected after 2000. However, we still found qualitatively similar results (analyses not shown). Thus, the use of a unique intensity of urbanization for a specific year seemed not to be an issue for the analysis of the correlation between urbanization on breeding phenology. Indeed, even if European cities did not grow at the same speed, a highly urbanized city in mid‐century is also likely to be equally highly urbanized today (EEA 2015).

Laying date was not related to the intensity of urbanization in any of the four species. The lack of significant relationship between the intensity of urbanization and laying date in all four species could be due to lack of sensitivity to urbanization or due to the proxy, the intensity of urbanization, used to quantify the degree of human impact on the environment. According to the relationship between CORINE land cover code and the intensity of urbanization, the index measured was related to anthropogenization of areas although radius could still be too small for some borders of monitored study plots. Although we studied local urbanization of study plots, it is still possible that human impact affects the environment at larger scales (Bol'shakov et al. 2001; Pickett et al. 2011; Gil and Brumm 2013; Johnson et al. 2013; Vittoz et al. 2013). The findings could be affected by the lack of highly urbanized plots, because the plots sampled in our study did not cover all variation in the intensity of urbanization in all European countries. Data were limited by availability of boxes differing in extent of urbanization, although we consider that this is not a serious issue in the present study because rural plots included natural plots as forests and agricultural or industrial plots where people also live. Nevertheless, we have shown that laying dates of the four species were not related to the intensity of local urbanization and lack of data does not seem to affect this result.

Collared and pied flycatchers showed a significant negative relationship between the intensity of urbanization and clutch size. Migratory status could be the decisive factor for the decrease in clutch size in flycatchers and the absence of such a difference in tits. The lack of a significant effect in tits could also be due to differences in thermal capacity as blue and great tits live at more variable latitudes than collared and pied flycatchers (Fig. S3; Svensson 1992; Del Hoyo et al. 2007) and hence display a larger range of temperature tolerance. This is the first time that a negative relationship has been shown between clutch size and intensity of urbanization in the two long‐distance migratory flycatcher species (Both et al. 2004, 2006; Laaksonen et al. 2006; Mizuta 2006; Pulido 2007; Sisask et al. 2010; Massa et al. 2011; Smallegange et al. 2011). More studies are needed to understand the underlying mechanism of intensity of urbanization on clutch size, and it is even possible that a reduction in clutch size was due to a combination of local conditions (Pearson and Lack 1992; Both et al. 2006) determined by ambient temperature (Burrows et al. 2011; Stocker et al. 2013), artificial night light (Small and Elvidge 2011; Dominoni et al. 2014; Fonken and Nelson 2014), food availability (Stenning 1995; Robb et al. 2008a,b; Saggese et al. 2011), avian population density (Krebs 1970; Stenning et al. 1988), nest predation by cats (Zanette et al. 2011), or vandalism (Brahmia et al. 2013).

In conclusion, in this first large‐scale study of life history traits and intensity of urbanization we showed a complex species‐specific major relationship between intensity of urbanization and breeding. The underlying mechanisms for the relationships between life history and intensity of urbanization remain to be determined. However, we propose that effects of food abundance or quality, avian population density, temperature, noise, pollution or disturbance by humans may on their own or in combination affect laying date and/or clutch size. Experiments could compare main and interactive effects of bird feeding by humans (Chamberlain et al. 2009) and temperature increases on advances in breeding date in neighboring urban and rural habitats.

## Conflict of Interest

None declared.

## Supporting information


**Figure S1**. Intensity of urbanisation according to (A) classification by scientists. Box plots show medians, quartiles, 5‐ and 95‐percentiles, and extreme values, and (B) CORINE land cover code (red = discontinuous urban, purple = industrial or commercial units, pink = green urban sites, brown = arable land and rice field, orange = agriculture lands, green = forest and natural field and blue = inland marshes).

**Figure S2.** Distribution of study plots across Europe, North Africa and the Middle East.

**Figure S3.** Box plots of latitude of study plots in four passerine birds in Europe, North Africa and the Middle East.

**Table S1.** Summary data for study plots. See Material and methods for definitions.
**Table S2.** Correlation matrix of explanatory variables.
**Table S3.** Mixed linear model investigating laying date in four passerines species (CF: Collared Flycatcher, GT: Great tit and PF: Pied Flycatcher) as a function of habitat characteristics (intensity of urbanisation, latitude, latitude squared, longitude, longitude squared, altitude (log‐transformed), and dominant habitat), nest box characteristics (nest floor surface and nest box material) and year as fixed effects, with study plot as a random factor.
