# Supplementary material for: Interspecific variation in the relationship between clutch size, laying date and intensity of urbanization in four species of hole‐nesting birds
Source: Ecol Evol. 2016 Jul 25;6(16):5907–20. doi: 10.1002/ece3.2335 (PMC4983601; doi:10.1002/ece3.2335)

Figure S1. Intensity of urbanisation according to A) classification by scientists. Box plots show medians, quartiles, 5- and 95-percentiles, and extreme values, and B) CORINE land cover code (red = discontinuous urban, purple = industrial or commercial units, pink = green urban sites, brown = arable land and rice field, orange = agriculture lands, green = forest and natural field and blue = inland marshes). Mean ± SE are shown and number of study plots is added to the figure (N = 191).

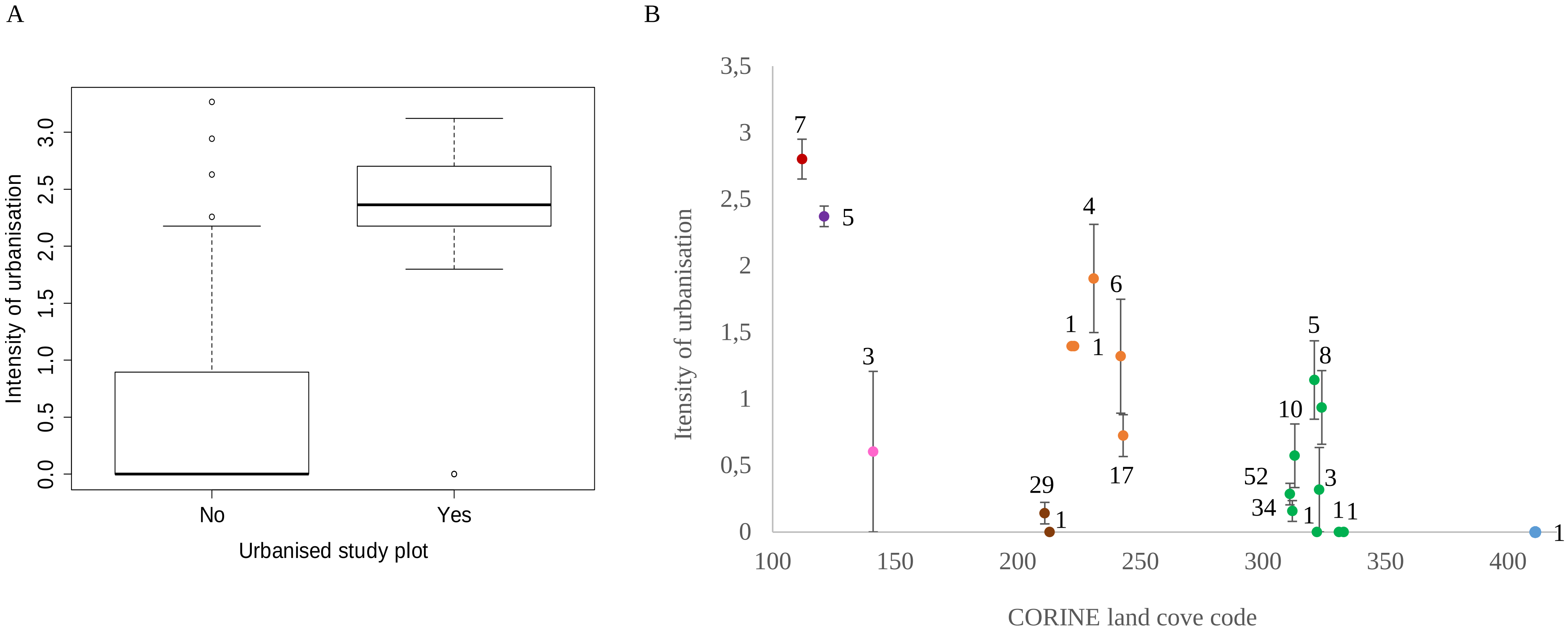

Supplement: Supplementary file 1 — Figure S1. Intensity of urbanisation according to (A) classification by scientists. Box plots show medians, quartiles, 5‐ and 95‐percentiles, and extreme values, and (B) CORINE land cover code (red = discontinuous urban, purple = industrial or commercial units, pink = green urban sites, brown = arable land and rice field, orange = agriculture lands, green = forest and natural field and blue = inland marshes). [file ECE3-6-5907-s001.pdf]
