# Supplementary material for: Interspecific variation in the relationship between clutch size, laying date and intensity of urbanization in four species of hole‐nesting birds
Source: Ecol Evol. 2016 Jul 25;6(16):5907–20. doi: 10.1002/ece3.2335 (PMC4983601; doi:10.1002/ece3.2335)

**Figure S2.** Distribution of study plots across Europe, North Africa and the Middle East. Blue plots are subsample plots used to estimate the difference in intensity of urbanisation between radius of 200, 500 and 1000 m.


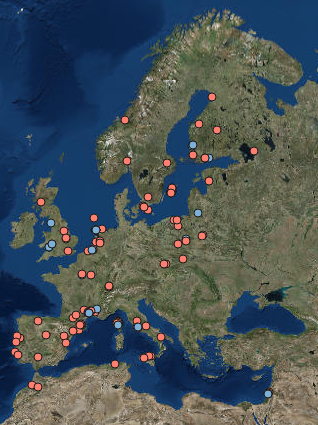

Supplement: Supplementary file 2 — Figure S2. Distribution of study plots across Europe, North Africa and the Middle East. [file ECE3-6-5907-s002.docx]
