# Supplementary material for: Interspecific variation in the relationship between clutch size, laying date and intensity of urbanization in four species of hole‐nesting birds
Source: Ecol Evol. 2016 Jul 25;6(16):5907–20. doi: 10.1002/ece3.2335 (PMC4983601; doi:10.1002/ece3.2335)

**Figure S3.** Box plots of latitude of study plots in four passerine birds in Europe, North Africa and the Middle East. Box plots show medians, quartiles, 5- and 95-percentiles, and extreme values.


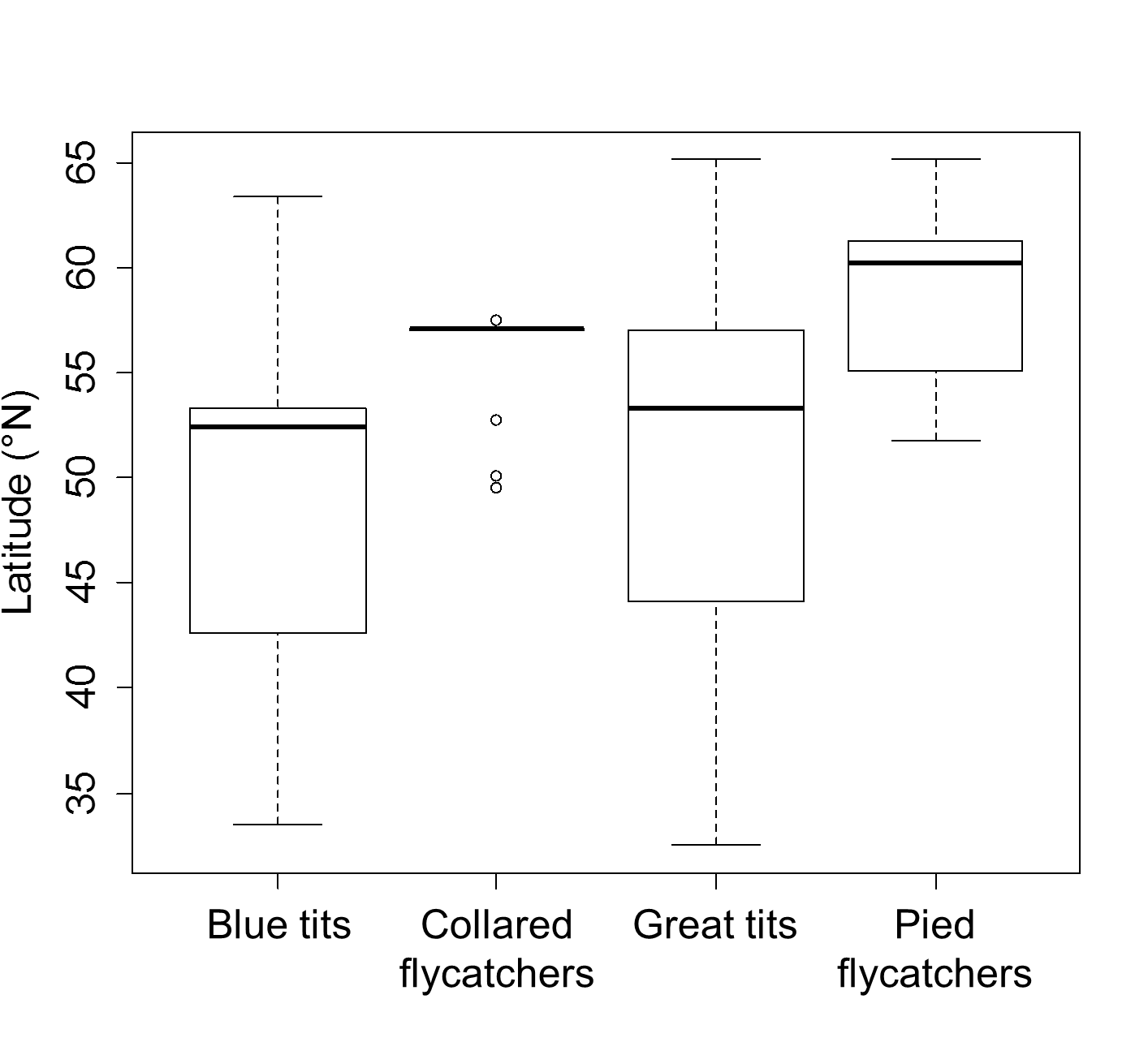

Supplement: Supplementary file 3 — Figure S3. Box plots of latitude of study plots in four passerine birds in Europe, North Africa and the Middle East. [file ECE3-6-5907-s003.docx]
