# Supplementary material for: Interspecific variation in the relationship between clutch size, laying date and intensity of urbanization in four species of hole‐nesting birds
Source: Ecol Evol. 2016 Jul 25;6(16):5907–20. doi: 10.1002/ece3.2335 (PMC4983601; doi:10.1002/ece3.2335)
Supplement: Supplementary file 4 — Table S1. Summary data for study plots. See Material and methods for definitions. Table S2. Correlation matrix of explanatory variables. Table S3. Mixed linear model investigating laying date in four passerines species (CF: Collared Flycatcher, GT: Great tit and PF: Pied Flycatcher) as a function of habitat characteristics (intensity of urbanisation, latitude, latitude squared, longitude, longitude squared, altitude (log‐transformed), and dominant habitat), nest box characteristics (nest floor surface and nest box material) and year as fixed effects, with study plot as a random factor. [file ECE3-6-5907-s004.docx]

**Supplementary material**

**Table S1.** Summary data for study plots. See Material and methods for definitions.

| Study site | BT | CF | GT | PF | TestDist | Latitude (°N) | Longitude (°E) | Altitude | Area | Urbanised. | Houses/km² | Intensity of urbanisation | Exact location |
| --- | --- | --- | --- | --- | --- | --- | --- | --- | --- | --- | --- | --- | --- |
| Alviano | no | no | yes | no | no | 42.622347 | 12.24265 | 90 | Italy | no | 0 | 0 | yes |
| Arinelle | yes | no | yes | no | no | 42.5920178 | 8.96317587 | 80 | Corsica | no | 15.9154943 | 1.22828469 | yes |
| Aulnaie | yes | no | yes | no | yes | 42.420917 | 8.665214 | 0 | Corsica | no | 0 | 0 | yes |
| Avapessa | yes | no | yes | no | no | 42.5552964 | 8.8970414 | 350 | Corsica | no | 0 | 0 | yes |
| Barcelona | no | no | yes | no | no | 41.46454414 | 2.143986 | 225 | Spain | no | 0 | 0 | yes |
| Bern-Bremer | no | no | yes | no | no | 46.961333 | 7.406778 | 558 | Switzerland | no | 0 | 0 | yes |
| Bern-Forst | no | no | yes | no | no | 46.918611 | 7.3105 | 650 | Switzerland | no | 55.7042301 | 1.75361546 | yes |
| Bern-Köniz | no | no | yes | no | no | 46.931972 | 7.401639 | 640 | Switzerland | no | 0 | 0 | yes |
| Bern-Spielwald | no | no | yes | no | no | 46.947778 | 7.315833 | 650 | Switzerland | no | 0 | 0 | yes |
| Białowieża (primeval stands) | no | yes | no | no | no | 52.718176 | 23.850308 | 160 | Poland | no | 0 | 0 | yes |
| Białowieża CZ (managed stands) | no | no | no | yes | no | 52.672407 | 23.724228 | 160 | Poland | no | 0 | 0 | yes |
| BOSHOEK | yes | no | yes | no | yes | 51.1331 | 4.519 | 10 | Belgium | no | 15.9154943 | 1.22828469 | yes |
| Brabtia | yes | no | no | no | no | 36.851797 | 8.331434 | 30 | Algeria | no | 55.7042301 | 1.75361546 | yes |
| Brampton Wood | no | no | yes | no | no | 52.313056 | -0.266667 | 35 | England | no | 0 | 0 | yes |
| Bussaco | yes | no | yes | no | no | 40.371733 | -8.366527 | 500 | Portugal | no | 0 | 0 | yes |
| Bute Park. Cardiff | yes | no | yes | no | yes | 51.486389 | -3.183333 | 8 | Wales | yes | 183.028185 | 2.26488434 | yes |
| Buunderkamp | no | no | no | yes | no | 52 | 5 | 100 | Netherlands | no | 7.95774715 | 0.9521988 | no |
| Caltavuturo | no | no | yes | no | no | 37.79 | 13.9139 | 800 | Sicily | no | 55.7042301 | 1.75361546 | yes |
| Cantabrian mountains | yes | no | yes | no | no | 42.639967 | -5.093158 | 995 | Spain | no | 0 | 0 | yes |
| Cap Corse | yes | no | no | no | yes | 42.357819 | 8.755428 | 900 | Corsica | no | 0 | 0 | yes |
| Castelporziano | yes | no | yes | no | no | 41.722199 | 12.408438 | 0 | Italy | no | 0 | 0 | yes |
| Cesarò | yes | no | yes | no | no | 37.952075 | 14.693217 | 1500 | Sicily | no | 0 | 0 | yes |
| Corte | yes | no | no | no | no | 42.280308 | 9.203445 | 550 | Corsica | no | 39.7887358 | 1.61054024 | yes |
| Cyncoed | yes | no | yes | no | yes | 51.519722 | -3.163056 | 65 | Wales | yes | 1066.33812 | 3.02830202 | yes |
| Dæli. Bærum | no | no | no | yes | no | 59.9321 | 10.5490 | 161 | Norway | no | 39.7887358 | 1.61054024 | yes |
| Dunas São Jacinto | no | no | yes | no | no | 40.680928 | -8.729632 | 8 | Portugal | no | 0 | 0 | yes |
| El Pedroso | yes | no | no | no | no | 37.78 | -5.08 | 500 | Spain | no | 23.8732415 | 1.39573239 | no |
| El Titllar | no | no | yes | no | no | 41.337968 | 1.015828 | 925 | Spain | no | 0 | 0 | yes |
| Feliceto | yes | no | yes | no | no | 42.5480642 | 8.93379121 | 280 | Corsica | no | 31.8309886 | 1.51628396 | yes |
| Filagna | yes | no | yes | no | yes | 42.5869699 | 8.96154104 | 100 | Corsica | no | 39.7887358 | 1.61054024 | yes |
| Finestres | yes | no | yes | no | no | 42.112864 | 2.599039 | 650 | Spain | no | 0 | 0 | yes |
| Foljuif-Nemours | yes | no | yes | no | yes | 48.288158 | 2.674152 | 96 | France | no | 31.8309886 | 1.51628396 | yes |
| Fontainebleau | yes | no | yes | no | no | 48.421965 | 2.678144 | 95 | France | no | 0 | 0 | yes |
| Forêt d'Orient | yes | no | yes | no | no | 48.284424 | 4.252841 | 150 | France | no | 0 | 0 | yes |
| Genovesa | yes | no | yes | no | no | 42.5250 | 8.858333 | 150 | Corsica | no | 0 | 0 | yes |
| GENT | yes | no | yes | no | no | 51.0051 | 3.7000 | 10 | Belgium | no | 23.8732415 | 1.39573239 | yes |
| Golani | no | no | yes | no | no | 32.781813 | 35.419008 | 213 | Israel | no | 15.9154943 | 1.22828469 | yes |
| Gole del Sagittario | yes | no | yes | no | no | 41.982026 | 13.801916 | 600 | Italy | no | 0 | 0 | yes |
| Gotland-AL | no | yes | yes | no | no | 57.098096 | 18.313756 | 6 | Sweden | no | 0 | 0 | yes |
| Gotland-AN | yes | yes | yes | no | no | 57.084266 | 18.314587 | 6 | Sweden | no | 0 | 0 | yes |
| Gotland-BF | no | yes | no | no | no | 57.210402 | 18.329574 | 6 | Sweden | no | 0 | 0 | yes |
| Gotland-BH | no | yes | yes | no | no | 57.111941 | 18.384268 | 6 | Sweden | no | 0 | 0 | yes |
| Gotland-BI1 | no | yes | no | no | no | 57.191904 | 18.366889 | 6 | Sweden | no | 0 | 0 | yes |
| Gotland-BI2 | no | yes | no | no | no | 57.197474 | 18.354555 | 6 | Sweden | no | 0 | 0 | yes |
| Gotland-BJ | yes | yes | yes | no | no | 57.056293 | 18.299668 | 6 | Sweden | no | 7.95774715 | 0.9521988 | yes |
| Gotland-BK | no | yes | yes | no | no | 57.037613 | 18.290222 | 6 | Sweden | no | 0 | 0 | yes |
| Gotland-BO | yes | yes | yes | no | no | 57.018669 | 18.280267 | 6 | Sweden | no | 7.95774715 | 0.9521988 | yes |
| Gotland-BP | no | no | yes | no | no | 57.5 | 18.5 | 6 | Sweden | no | 0 | 0 | no |
| Gotland-BS | yes | yes | yes | no | no | 57.052075 | 18.292604 | 6 | Sweden | no | 7.95774715 | 0.9521988 | yes |
| Gotland-BU | no | yes | no | no | no | 57.5 | 18.5 | 6 | Sweden | no | 0 | 0 | no |
| Gotland-DO | no | yes | yes | no | no | 57.109204 | 18.340844 | 6 | Sweden | no | 0 | 0 | yes |
| Gotland-DT | no | yes | yes | no | no | 57.128568 | 18.314912 | 6 | Sweden | no | 0 | 0 | yes |
| Gotland-ET | no | yes | no | no | no | 57.5 | 18.5 | 6 | Sweden | no | 0 | 0 | no |
| Gotland-FA | yes | yes | yes | no | no | 57.003535 | 18.326503 | 6 | Sweden | no | 39.7887358 | 1.61054024 | yes |
| Gotland-FE | no | yes | no | no | no | 57.5 | 18.5 | 6 | Sweden | no | 0 | 0 | no |
| Gotland-FG | no | yes | no | no | no | 57.5 | 18.5 | 6 | Sweden | no | 0 | 0 | no |
| Gotland-FK | yes | yes | yes | no | no | 57.070224 | 18.305909 | 6 | Sweden | no | 0 | 0 | yes |
| Gotland-FL | no | yes | no | no | no | 57.5 | 18.5 | 6 | Sweden | no | 0 | 0 | no |
| Gotland-FO | yes | yes | yes | no | no | 57.080036 | 18.303279 | 6 | Sweden | no | 0 | 0 | yes |
| Gotland-FP | yes | yes | yes | no | no | 57.084182 | 18.306161 | 6 | Sweden | no | 0 | 0 | yes |
| Gotland-GA | no | yes | yes | no | no | 57.14732 | 18.305243 | 6 | Sweden | no | 0 | 0 | yes |
| Gotland-GB | no | yes | yes | no | no | 57.135377 | 18.334435 | 6 | Sweden | no | 0 | 0 | yes |
| Gotland-GE | no | yes | no | no | no | 57.5 | 18.5 | 6 | Sweden | no | 0 | 0 | no |
| Gotland-GO | no | yes | no | no | no | 57.5 | 18.5 | 6 | Sweden | no | 0 | 0 | no |
| Gotland-GR | yes | yes | yes | no | no | 57.058096 | 18.322457 | 6 | Sweden | no | 0 | 0 | yes |
| Gotland-GU | no | yes | no | no | no | 57.172942 | 18.400334 | 6 | Sweden | no | 0 | 0 | yes |
| Gotland-HA | no | yes | no | no | no | 57.5 | 18.5 | 6 | Sweden | no | 0 | 0 | no |
| Gotland-HG | no | yes | no | no | no | 57.21328 | 18.250019 | 6 | Sweden | no | 0 | 0 | yes |
| Gotland-HM | no | yes | no | no | no | 57.5 | 18.5 | 6 | Sweden | no | 0 | 0 | no |
| Gotland-JA | no | yes | no | no | no | 57.209331 | 18.411273 | 6 | Sweden | no | 7.95774715 | 0.9521988 | yes |
| Gotland-JB | no | yes | no | no | no | 57.5 | 18.5 | 6 | Sweden | no | 0 | 0 | no |
| Gotland-JD | no | yes | no | no | no | 57.5 | 18.5 | 6 | Sweden | no | 0 | 0 | no |
| Gotland-JE | no | yes | no | no | no | 57.5 | 18.5 | 6 | Sweden | no | 0 | 0 | no |
| Gotland-JF | no | yes | no | no | no | 57.5 | 18.5 | 6 | Sweden | no | 0 | 0 | no |
| Gotland-JZ | no | yes | no | no | no | 57.5 | 18.5 | 6 | Sweden | no | 0 | 0 | no |
| Gotland-KA | no | yes | yes | no | no | 57.118656 | 18.344841 | 6 | Sweden | no | 0 | 0 | yes |
| Gotland-KT | no | yes | yes | no | no | 57.034967 | 18.30657 | 6 | Sweden | no | 0 | 0 | yes |
| Gotland-LI | no | yes | yes | no | no | 57.137126 | 18.314163 | 6 | Sweden | no | 0 | 0 | yes |
| Gotland-LO | no | yes | no | no | no | 57.5 | 18.5 | 6 | Sweden | no | 0 | 0 | no |
| Gotland-OG | no | yes | no | no | no | 57.5 | 18.5 | 6 | Sweden | no | 0 | 0 | no |
| Gotland-OJ | yes | yes | yes | no | no | 57.041057 | 18.298337 | 6 | Sweden | no | 0 | 0 | yes |
| Gotland-OL | no | yes | yes | no | no | 57.003367 | 18.317148 | 6 | Sweden | no | 0 | 0 | yes |
| Gotland-RM | no | yes | yes | no | no | 57.080103 | 18.330074 | 6 | Sweden | no | 23.8732415 | 1.39573239 | yes |
| Gotland-RN | no | yes | yes | no | no | 57.010469 | 18.31792 | 6 | Sweden | no | 0 | 0 | yes |
| Gotland-RO | yes | yes | yes | no | no | 57.033973 | 18.294481 | 6 | Sweden | no | 31.8309886 | 1.51628396 | yes |
| Gotland-RUE | yes | yes | yes | no | no | 57.015129 | 18.308991 | 6 | Sweden | no | 7.95774715 | 0.9521988 | yes |
| Gotland-RUW | yes | yes | yes | no | no | 57.019404 | 18.296045 | 6 | Sweden | no | 0 | 0 | yes |
| Gotland-SA | no | yes | no | no | no | 57.5 | 18.5 | 6 | Sweden | no | 0 | 0 | no |
| Gotland-SB | no | yes | yes | no | no | 57.028548 | 18.286096 | 6 | Sweden | no | 71.6197244 | 1.8610546 | yes |
| Gotland-SI | no | yes | no | no | no | 57.1836 | 18.445354 | 6 | Sweden | no | 15.9154943 | 1.22828469 | yes |
| Gotland-SJ | no | yes | no | no | no | 57.5 | 18.5 | 6 | Sweden | no | 0 | 0 | no |
| Gotland-SL | no | yes | yes | no | no | 57.105283 | 18.372455 | 6 | Sweden | no | 0 | 0 | yes |
| Gotland-SP | no | yes | no | no | no | 57.5 | 18.5 | 6 | Sweden | no | 0 | 0 | no |
| Gotland-ST | no | yes | no | no | no | 57.5 | 18.5 | 6 | Sweden | no | 0 | 0 | no |
| Gotland-SU | no | yes | yes | no | no | 57.124186 | 18.324364 | 6 | Sweden | no | 0 | 0 | yes |
| Gotland-SV | no | yes | no | no | no | 57.169183 | 18.267104 | 6 | Sweden | no | 0 | 0 | yes |
| Gotland-TB | no | yes | yes | no | no | 57.075534 | 18.337081 | 6 | Sweden | no | 0 | 0 | yes |
| Gotland-TU | yes | yes | yes | no | no | 57.087361 | 18.336725 | 6 | Sweden | no | 0 | 0 | yes |
| Gotland-UH | no | yes | no | no | no | 57.5 | 18.5 | 6 | Sweden | no | 0 | 0 | no |
| Gotland-VA | no | yes | no | no | no | 57.5 | 18.5 | 6 | Sweden | no | 0 | 0 | no |
| Gotland-VL | no | yes | yes | no | no | 57.020245 | 18.220089 | 6 | Sweden | no | 0 | 0 | yes |
| Grassa | yes | no | no | no | no | 42.5879015 | 8.9726958 | 150 | Corsica | no | 0 | 0 | yes |
| Grobla | yes | yes | yes | no | no | 50.112507 | 20.420823 | 190 | Poland | no | 0 | 0 | yes |
| Grygov-Olomouc | yes | yes | yes | no | no | 49.51591667 | 17.3002778 | 215 | Czech Republic | no | 0 | 0 | yes |
| Harjavalta Rural | yes | no | yes | yes | yes | 61.250113 | 22.003074 | 55 | Finland | no | 47.7464829 | 1.68794329 | yes |
| Harjavalta Urban | yes | no | yes | yes | yes | 61.327301 | 22.111246 | 55 | Finland | yes | 230.774667 | 2.36506597 | yes |
| Heath | yes | no | no | no | yes | 51.513889 | -3.188333 | 65 | Wales | yes | 254.647909 | 2.40764224 | yes |
| Herdade da Apostiça | no | no | yes | no | no | 38.5370 | -9.0990 | 15 | Portugal | no | 0 | 0 | yes |
| Herdade da Ribeira de Baixo | yes | no | yes | no | no | 38.10991 | -8.58863 | 210 | Portugal | no | 15.9154943 | 1.22828469 | yes |
| Hoge VeluweA | no | no | yes | no | no | 52.1 | 5.78 | 100 | Netherlands | no | 71.6197244 | 1.8610546 | no |
| Jordà | yes | no | yes | no | no | 42.149739 | 2.515906 | 560 | Spain | no | 0 | 0 | yes |
| Kauhava | no | no | yes | no | no | 63.083321 | 23.233339 | 75 | Finland | no | 0 | 0 | yes |
| Kfar Hahoresh | no | no | yes | no | no | 32.700556 | 35.265525 | 389 | Israel | no | 55.7042301 | 1.75361546 | yes |
| Konnevesi | no | no | yes | yes | no | 62.6055988 | 26.3446676 | 100 | Finland | no | 15.9154943 | 1.22828469 | yes |
| Ladoga (Mayachino) | no | no | yes | yes | no | 60.779404 | 32.823372 | 10 | Russia | no | 0 | 0 | yes |
| Lake wood. Sussex | yes | no | no | no | no | 50.976419 | 0.0822 | 120 | England | no | 23.8732415 | 1.39573239 | yes |
| Lancaster | yes | no | no | no | yes | 54.010635 | -2.778307 | 50 | England | no | 71.6197244 | 1.8610546 | yes |
| Lauwersmeer | no | no | yes | no | no | 53.4 | 6.2 | 3 | Netherlands | no | 0 | 0 | no |
| Le Bruguier | no | no | yes | no | no | 44 | 3 | 250 | France | no | 0 | 0 | no |
| Liesbos | yes | no | yes | no | no | 54.58 | 4.67 | 5 | Netherlands | no | 183.028185 | 2.26488434 | no |
| Lincoln | yes | no | yes | no | no | 53.2 | -0.59 | 30 | England | no | 429.718346 | 2.63419337 | no |
| Liouc | yes | no | yes | no | no | 43.890161 | 3.999778 | 100 | France | no | 15.9154943 | 1.22828469 | yes |
| Lodz. Lagiewniki Forest | yes | no | yes | no | no | 51.8406275 | 19.4896166 | 230 | Poland | no | 0 | 0 | yes |
| MacchiaGrande-Orbetello-Burano | no | no | yes | no | no | 41.829706 | 12.211638 | 0 | Italy | no | 0 | 0 | yes |
| Mal | yes | no | no | no | no | 42.5 | 8.9 | 500 | Corsica | no | 0 | 0 | no |
| Mamora | yes | no | no | no | no | 33.8 | -6.1 | 50 | Marocco | no | 95.4929659 | 1.98449566 | no |
| Manferrara | yes | no | yes | no | no | 40.53755 | 16.506565 | 454 | Italy | no | 15.9154943 | 1.22828469 | yes |
| Mata Nacional de Leiria | no | no | yes | no | no | 39.8530 | -8.9440 | 25 | Portugal | no | 0 | 0 | yes |
| Megido | no | no | yes | no | yes | 32.521311 | 35.33618 | 218 | Israel | no | 0 | 0 | yes |
| Miraflores de la Sierra | yes | no | no | no | no | 40.811359 | -3.786641 | 1200 | Spain | no | 0 | 0 | yes |
| Mistretta | yes | no | yes | no | no | 37.953001 | 14.414274 | 1070 | Sicily | no | 0 | 0 | yes |
| Mlociny | yes | no | yes | no | yes | 52.315054 | 20.926634 | 80 | Poland | yes | 0 | 0 | yes |
| Mollégès | no | no | yes | no | no | 43.805833 | 4.949444 | 55 | France | no | 1838.23959 | 3.26463831 | yes |
| Monks Wood. Cambridgeshire | yes | no | yes | no | no | 52.409722 | -0.236111 | 26 | England | no | 0 | 0 | yes |
| Montpellier-CEFE | no | no | yes | no | yes | 43.637983 | 3.862286 | 60 | France | yes | 501.338071 | 2.70099609 | yes |
| Montpellier-city | yes | no | yes | no | yes | 43.640564 | 3.876383 | 60 | France | yes | 151.197196 | 2.18240665 | yes |
| Montpellier-Jardin des Plantes | no | no | yes | no | yes | 43.614436 | 3.871694 | 60 | France | yes | 851.478946 | 2.93068366 | yes |
| Montpellier-University | no | no | yes | no | yes | 43.632356 | 3.866394 | 60 | France | yes | 230.774667 | 2.36506597 | yes |
| Montseny | yes | no | yes | no | no | 42.304111 | 2.432228 | 1150 | Spain | no | 0 | 0 | yes |
| Moshav Ram-On-Garden | no | no | yes | no | yes | 32.526764 | 35.2599 | 85 | Israel | yes | 501.338071 | 2.70099609 | yes |
| Moulis | yes | no | yes | no | no | 42.96742 | 1.092041 | 500 | France | no | 0 | 0 | yes |
| Murato | yes | no | yes | no | no | 42.551883 | 8.91815 | 280 | Corsica | no | 151.197196 | 2.18240665 | yes |
| Muro | yes | no | yes | no | yes | 42.5510816 | 8.92442026 | 280 | Corsica | no | 55.7042301 | 1.75361546 | yes |
| Near Uppsala | no | no | no | yes | no | 59.78 | 17.59 | 20 | Sweden | no | 0 | 0 | no |
| Nagshead Plantation | yes | no | yes | yes | no | 51.777752 | -2.573603 | 150 | England | no | 0 | 0 | yes |
| Oosterhout | no | no | no | yes | no | 51.92 | 5.83 | 10 | Netherlands | no | 875.352187 | 2.94267867 | no |
| Nagshead. Gloucestershire | no | no | no | yes | yes | 51.777752 | -2.573603 | 150 | England | no | 0 | 0 | yes |
| Near Kilingi-NõmmeConiferous | no | no | yes | yes | no | 58.139042 | 24.922201 | 70 | Estonia | no | 15.9154943 | 1.22828469 | yes |
| Near Kilingi-NõmmeDeciduous | no | no | yes | yes | no | 58.159413 | 25.00586 | 70 | Estonia | no | 0 | 0 | yes |
| Olmi | yes | no | no | no | yes | 42.533364 | 9.030659 | 1000 | Corsica | no | 0 | 0 | yes |
| Oulu | yes | no | yes | no | no | 65.108889 | 25.542787 | 50 | Finland | no | 0 | 0 | yes |
| Pacciani | yes | no | no | no | no | 42.516667 | 8.861111 | 180 | Corsica | no | 23.8732415 | 1.39573239 | yes |
| PEERDSBOS | yes | no | yes | no | no | 51.2725 | 4.4884 | 10 | Belgium | no | 0 | 0 | yes |
| Pietra | yes | no | yes | no | no | 42.522222 | 8.883333 | 180 | Corsica | no | 31.8309886 | 1.51628396 | yes |
| Pioggiola | yes | no | no | no | yes | 42.542086 | 8.99308 | 500 | Corsica | no | 0 | 0 | yes |
| Pirio | yes | no | yes | no | yes | 42.3764627 | 8.7492539 | 200 | Corsica | no | 55.7042301 | 1.75361546 | yes |
| Pitarque | no | no | yes | no | no | 40.653067 | -0.618194 | 1000 | Spain | no | 7.95774715 | 0.9521988 | yes |
| Ponte | yes | no | yes | no | no | 42.523611 | 8.873611 | 180 | Corsica | no | 7.95774715 | 0.9521988 | yes |
| Ras El Ma. Ifrane | yes | no | no | no | no | 33.5 | -5.1 | 1650 | Middle Atlas | no | 0 | 0 | no |
| Prepirineu | yes | no | yes | no | no | 42.116767 | 2.339545 | 1120 | Spain | no | 0 | 0 | yes |
| Prezunna | yes | no | no | no | no | 42.431955 | 8.757384 | 300 | Corsica | no | 23.8732415 | 1.39573239 | yes |
| Puéchabon | yes | no | yes | no | no | 43.740756 | 3.597133 | 160 | France | no | 0 | 0 | yes |
| Rowardennan | yes | no | yes | yes | no | 56.13 | -4.61 | 8 | Scotland | no | 7.95774715 | 0.9521988 | no |
| Quissac | yes | no | yes | no | no | 43.926933 | 3.986767 | 100 | France | no | 119.366207 | 2.08050458 | yes |
| Riudabella | no | no | yes | no | no | 41.376366 | 1.059687 | 500 | Spain | no | 0 | 0 | yes |
| Rome | yes | no | no | no | yes | 41.913759 | 12.486238 | 50 | Italy | yes | 63.6619772 | 1.81064898 | yes |
| Rouvière | yes | no | yes | no | no | 43.665528 | 3.669706 | 260 | France | no | 0 | 0 | yes |
| Ruissalo | yes | no | yes | yes | no | 60.434707 | 22.178758 | 10 | Finland | no | 0 | 0 | yes |
| Ruskeasuo. Helsinki | yes | no | yes | no | yes | 60.210267 | 24.912396 | 50 | Finland | yes | 0 | 0 | yes |
| Siuntio. Plot A | no | no | no | yes | no | 60.25 | 24.3 | 75 | Finland | no | 0 | 0 | no |
| Siuntio. Plot B | no | no | no | yes | no | 60.25 | 24.3 | 75 | Finland | no | 0 | 0 | no |
| Sagunto | no | no | yes | no | no | 39.704315 | -0.232665 | 30 | Spain | no | 23.8732415 | 1.39573239 | yes |
| Sakiai | yes | no | yes | yes | yes | 55.053387 | 23.070427 | 55 | Lituania | no | 0 | 0 | yes |
| Santo Stefano di Quisquina | yes | no | yes | no | no | 37.6111 | 13.5387 | 960 | Sicily | no | 0 | 0 | yes |
| Sekocin | no | no | yes | yes | no | 52.097189 | 20.877627 | 120 | Poland | no | 0 | 0 | yes |
| Sobieszewo Island | no | no | yes | no | no | 54.348289 | 18.845543 | 13 | Poland | no | 0 | 0 | yes |
| South Skåne. Hagestad | yes | no | yes | no | no | 55.385312 | 14.151648 | 10 | Sweden | no | 0 | 0 | yes |
| South Skåne. Kungsmarken | no | no | yes | no | no | 55.724291 | 13.27748 | 10 | Sweden | no | 0 | 0 | yes |
| South Skåne. Revinge | yes | no | no | no | no | 55.692315 | 13.465092 | 10 | Sweden | no | 7.95774715 | 0.9521988 | yes |
| Stenbrohult | yes | no | yes | yes | no | 56.612798 | 14.190506 | 140 | Sweden | no | 7.95774715 | 0.9521988 | yes |
| Sud Toulouse | yes | no | yes | no | no | 43.370418 | 1.559872 | 220 | France | no | 7.95774715 | 0.9521988 | yes |
| Szwalewo Forest | no | no | no | yes | no | 53.7190 | 19.5200 | 120 | Poland | no | 0 | 0 | yes |
| TaveraCB | yes | no | yes | no | yes | 42.069925 | 8.989228 | 465 | Corsica | no | 23.8732415 | 1.39573239 | yes |
| TaveraCV | yes | no | yes | no | yes | 42.0728 | 9.008436 | 465 | Corsica | no | 15.9154943 | 1.22828469 | yes |
| Trondheim. four study plots | yes | no | no | no | no | 63.346842 | 10.231611 | 60 | Norway | no | 0 | 0 | yes |
| Velky Kosir Hill | no | yes | yes | no | no | 49.54293333 | 17.0558194 | 350 | Czech Republic | no | 0 | 0 | yes |
| Ventoux | yes | no | yes | no | yes | 44.133333 | 5.183333 | 925 | France | no | 0 | 0 | yes |
| Vic | yes | no | yes | no | no | 43.866667 | 4.233333 | 100 | France | no | 0 | 0 | yes |
| Vistula Spit | no | no | no | yes | no | 54.3600 | 19.3470 | 10 | Poland | no | 0 | 0 | yes |
| Vlieland | no | no | yes | no | no | 53.25 | 4.95 | 5 | Vlieland | no | 0 | 0 | no |
| Vlieland-51 | yes | no | yes | no | yes | 53.270975 | 4.978762 | 5 | Vlieland | no | 0 | 0 | yes |
| Vlieland-52 | yes | no | yes | no | no | 53.274957 | 4.997294 | 5 | Vlieland | no | 47.7464829 | 1.68794329 | yes |
| Vlieland-53 | yes | no | yes | no | yes | 53.279315 | 5.012188 | 5 | Vlieland | no | 0 | 0 | yes |
| Vlieland-54 | yes | no | yes | no | yes | 53.284576 | 5.026605 | 5 | Vlieland | no | 23.8732415 | 1.39573239 | yes |
| Vlieland-55 | yes | no | yes | no | no | 53.296788 | 5.05092 | 15 | Vlieland | no | 0 | 0 | yes |
| Vlieland-56 | yes | no | yes | no | no | 53.301916 | 5.07235 | 5 | Vlieland | no | 55.7042301 | 1.75361546 | yes |
| Vlieland-57 | yes | no | yes | no | yes | 53.296176 | 5.071437 | 5 | Vlieland | yes | 1305.07053 | 3.11596663 | yes |
| Vomb | no | no | no | yes | no | 55.651516 | 13.562402 | 10 | Sweden | no | 0 | 0 | yes |
| Wilrijk | yes | no | yes | no | yes | 51.15896 | 4.408329 | 10 | Belgium | yes | 159.154943 | 2.20454035 | yes |

**Table S2.** Correlation matrix of explanatory variables. A) Pearson test was used for numerical variables (intensity of urbanisation, years, nest floor area, altitude log transformed, latitude and longitude). B) A linear mixed model (function lme, package nlme & function Anova, package car, type III) with study plot as a random effect was used to test the relationship between dominant habitat (C = Coniferous, D = Deciduous, E = Evergreen and M = Mixed), nest box material (C = Concrete and W = Wood) or species (BT = blue tit, GT = great tit, CF = collared flycatcher and PF = pied flycatcher) and numerical variables (intensity of urbanisation, years, nest floor area, altitude log transformed, latitude and longitude). Significant *P*-values are shown in bold.

| A | Intensity of urbanisation | | | Years | | | Nest floor area | | | Altitude (log) | | |
| --- | --- | --- | --- | --- | --- | --- | --- | --- | --- | --- | --- | --- |
|  | r_Pearson_ | *t_df_* | *P* | r_Pearson_ | *t_df_* | *P* | r_Pearson_ | *t_df_* | *p* | r_Pearson_ | *t_df_* | *P* |
| Years | -0.08 | -4.90_3415_ | **<0.01** |  |  |  |  |  |  |  |  |  |
| Nest floor area | -0.02 | -0.89_3415_ | 0.37 | 0.23 | 13.88_3415_ | **<0.01** |  |  |  |  |  |  |
| Altitude (log) | -0.08 | -4.94_3415_ | **<0.01** | 0.18 | 10.75_3415_ | **<0.01** | 0.44 | 28.78_3415_ | **< 0.01** |  |  |  |
| Latitude | 0.04 | 2.43_3315_ | **0.02** | -0.08 | -4.86_3415_ | **<0.01** | -0.19 | -11.45_3415_ | **<0.01** | -0.66 | -51.73_3415_ | **<0.01** |
| Longitude | -0.14 | -8.29_3415_ | **<0.01** | 0.24 | 14.45_3415_ | **<0.01** | -0.05 | -2.66_3415_ | **0.01** | -0.13 | -7.87_3415_ | **<0.01** |

| B | Dominant habitat | | | Material | | | Species | | |
| --- | --- | --- | --- | --- | --- | --- | --- | --- | --- |
|  | Difference | *F_df_* | *P* | Difference | *F_df_* | *p* | Difference | *F_df_* | *P* |
| Intensity of urbanisation | C=D=E=M | 3.05_3,3215_ | 0.38 | C=W | 0.05_1,3217_ | 0.83 | CF< PF≤BT=GT | 51.41_3,3415_ | **<0.01** |
| Years | M≤C=E≤D | 9.55_3,3215_ | **0.03** | C=W | 0.27_1,3217_ | 0.60 | PF<GT<BT<CF | 49.65_3,3215_ | **<0.01** |
| Nest floor area | C=D=E=M | 1.48_3,3215_ | 0.69 | C<W | 20.92_1,3217_ | **<0.01** | BT=GT=CF=PF | 3.50_3,3215_ | 0.32 |
| Altitude (log) | M=D≤C≤E | 11.63_3,3215_ | **<0.01** | C=W | 0.60_1,3217_ | 0.44 | CF<GT<BT=PF | 138.56_3,3215_ | **<0.01** |
| Latitude | C≤D=M≤E | 234.91_3,3215_ | **<0.01** | C=W | 0.01_1,3217_ | 0.99 | BT≤GT≤CF≤PF | 55.08_3,3214_ | **<0.01** |
| Longitude | C=D=E=M | 1.63_3,3215_ | 0.65 | C=W | 0.01_1,3217_ | 0.99 | BT≤GT≤CF≤PF | 55.10_3,3215_ | **<0.01** |

**Table S3.** Mixed linear model investigating laying date in four passerines species (CF: Collared Flycatcher, GT: Great tit and PF: Pied Flycatcher) as a function of habitat characteristics (intensity of urbanisation, latitude, latitude squared, longitude, longitude squared, altitude (log-transformed), and dominant habitat), nest box characteristics (nest floor surface and nest box material) and year as fixed effects, with study plot as a random factor. Significant *P*-values in the final models are shown in bold. Likelihood Ratio Tests (LRT) were calculated for the random effect of study plot.

|  | *F_df_* | *P* | Estimate ± SE |
| --- | --- | --- | --- |
| Intensity of urbanisation | 1.96_1,197_ | 0.17 | -8.99 ± 6.42 |
| Species | 13.29_3, 3191_ | **<0.01** | -8.53 ± 16.69 (CF)  -9.65 ± 2.70 (GT)  9.75 ± 26.60 (PF) |
| Latitude | 5.11_1,3191_ | **0.02** | -4.65 ± 2.06 |
| Latitude ^ 2 | 5.84_1, 3191_ | **0.02** | 0.05 ± 0.02 |
| Longitude | 2.29_1,3191_ | 0.13 | -0.81 ± 0.54 |
| Longitude^2 | 28.50_1, 3191_ | **<0.01** | -0.05 ± 0.01 |
| Year | 170.19_1, 3191_ | **< 0.01** | -0.12 ± 0.01 |
| Altitude (log) | 1.84_1, 3191_ | 0.17 | 1.73 ± 1.27 |
| Nest floor area | 0.02_1, 3191_ | 0.89 | -0.01 ± 0.01 |
| Nest box material | 0.10_1,33191_ | 0.76 | -0.67 ± 2.19 (Wood) |
| Dominant habitat | 50.91_3, 3191_ | **< 0.01** | -8.57 ± 1.50 (Deciduous)  -5.34 ± 1.74 (Evergreen)  -3.98 ± 0.73 (Mixed) |
| Intensity of urbanisation × Latitude | 1.44_1, 3191_ | 0.23 | 0.15 ± 0.13 |
| Intensity of urbanisation × Species | 34.26_3, 3191_ | **<0.01** | -76.01 ± 1470.27(CF)  14.83 ± 2.59 (GT)  32.81 ± 24.23 (PF) |
| Latitude × Species | 16.81_3, 3191_ | **<0.01** | 0.54 ± 0.30 (CF)  0.20 ± 0.05 (GT)  0.15 ± 0.47 (PF) |
| Latitude × Longitude | 12.67_1, 3191_ | **< 0.01** | 0.04 ± 0.01 |
| Intensity of urbanisation × Latitude × Species | 35.83_3, 3191_ | **<0.01** | 1.32 ± 25.78 (CF)  -0.29 ± 0.05 (GT)  -0.54 ± 0.40 (PF) |
| Study spot | LRT: 1001.43 | **< 0.01** |  |
